# Does Nature Need Rights?

**DOI:** 10.1093/ojls/gqaf021

**Published:** 2025-06-15

**Authors:** Lael K Weis, Robert Mullins

**Keywords:** rights of nature, green constitutionalism, environmental constitutionalism, non-anthropocentric constitutionalism, ecocentrism, ecocentric duties

## Abstract

Rights of nature (RoN) appear to provide a promising alternative to anthropocentric environmental rights. But do they meet the demands of transformative green constitutionalist projects? This article addresses that question by examining the juridical dimensions of RoN. We draw on empirical studies of RoN laws to identify and examine the challenges of redeploying ‘rights’ and ‘legal personality’—concepts associated with liberal normative frameworks—in the service of green normative theory and its fundamental concern for ecological well-being. We reject the dominant rights-based paradigm, which locates the green potential of RoN laws in constituting nature as a rights-bearing legal subject, and we propose an alternative: the governance paradigm. Our alternative locates the green potential of RoN laws in reconfiguring authority relations and supports ecocentric legal frameworks instead of RoN: emphasising ecocentric values and duties instead of rights, and ecological community membership instead of legal personhood.

## 1. Introduction

Rights of nature (RoN) recognise ecosystems as rights-bearing legal subjects. According to proponents, they are a ‘transformative norm … that embeds human societies … within ecosystems’,^1^ emphasising ‘the well-being of all members of the biotic community’.^2^ They seek to reorient legal systems towards valuing nature for its own sake and not merely for its contribution to human well-being, and towards a view of humans as members of interdependent and holistic living systems. RoN thus offer an alternative to conventional anthropocentric environmental rights, such as human rights to a clean and healthy environment.

This article evaluates the promise of RoN for transformative green constitutionalist projects, which seek to reconfigure constitutional principles, institutions and norms to reflect the commitments of *green normative theory*: that is, a theory of value which holds that ecosystems have intrinsic (ie non-derivative or final) value,^3^ and that is committed to a holistic understanding of values and practices of valuing.^4^ In asking whether nature needs rights, we are therefore interested in examining whether recognising nature as a rights-bearing legal subject is necessary—or, even if not strictly necessary, then desirable—for advancing green norms. By ‘green norms’, we mean norms that treat ecosystems as bearers of intrinsic value, reflect holistic understandings of value and promote practices of valuing that reflect these commitments. Are RoN transformative, as proponents suggest? Can they shift environmental governance away from a person–property paradigm that treats ecosystems as bundles of resources for human well-being? Do they help reconfigure the exercise of political power, however incrementally, to better reflect green normativity?

This article’s main contribution to these debates lies in conceptual modelling. It addresses these questions by critically examining the current rights-based paradigm used to model ‘RoN laws’—the term we use to refer to the set of laws typically referred to as ‘rights of nature’. Although we conclude that at least some of these laws are not best understood as rights-based legal frameworks (and, in this respect, ‘RoN laws’ is something of a misnomer), we adopt this terminology given its widespread use in the literature. In addition, in reaching this conclusion, the article makes a second contribution to these debates by introducing the idea of ‘ecocentric duties’ as a type of ‘intrinsic duty’: duties based on ecological community membership that are *not* derived from or justified by rights.^5^

Ultimately, we argue that the rights-based paradigm is not fit for green constitutionalist purposes. We propose an alternative approach, the governance paradigm, and suggest that green constitutionalists adopt ecocentric legal frameworks as an alternative model to RoN. Our proposal better captures the features of current RoN laws that have proven to be the most promising for advancing green normativity. Although ‘rights’ and ‘legal personhood’ are devices used in many of these legal regimes, they are neither central to how they advance green normativity in practice and nor are they particularly effective. The rights-based paradigm thus simultaneously distorts and undersells the green normative potential of these laws. We concede that ‘rights talk’ is often effective for mobilisation and gaining public support, as evidenced by the success of RoN norm entrepreneurs.^6^ However, we argue that green constitutionalists cannot properly evaluate these benefits without a sound conceptual model.

In advancing these arguments, our analysis focuses on the juridical dimensions of RoN laws: that is, we examine their capacity to reshape the understandings of legal actors in a manner that reflects the commitments of green normative theory, and not their instrumental utility in achieving environmental outcomes. Our analysis and proposed alternative build upon the qualitative empirical work of existing RoN scholarship, which details the practical operation of existing RoN laws. Our analysis and proposal are thus both indebted to and intended to complement this work, highlighting the importance of scholarly collaboration between empirically oriented and legal theoretic approaches.^7^ Drawing on these studies, we identify and critically examine the challenges of redeploying ‘rights’ and ‘legal personhood’—legal forms associated with liberal normative frameworks—to advance the transformative aims of green constitutionalism.

We begin by explaining the commitments of green normative theory that underpin the green constitutionalist project and situating our project within the literature (section 2). Next, we explain our research method and describe the current rights-based paradigm, which holds that the key to advancing green normativity lies in recognising nature as a rights-bearing legal subject (section 3). Within this paradigm, there are two conceptual models: the Nature’s Rights model and the Legal Personhood model. The Nature’s Rights model creates new rights, giving existing legal persons standing to bring lawsuits on behalf of nature’s interests. The Legal Personhood model creates new legal persons, appointing legal guardians to act on their behalf.

We then turn to a juridical analysis of RoN (section 4). Evaluating whether nature *needs* rights, we argue, requires attending to the justificatory structure of legal rights as such, as well as contending with dimensions of their judicialisation. This includes the paradigmatic adjudicative function of rights in liberal constitutional systems as a mechanism for justifying state action in light of competing interests. Here we suggest that debates among rights theorists—which focus on whether nature *can* hold rights—helpfully inform this question by drawing attention to the consequences of this adjudicative function. Overall, these debates suggest that a rights-based paradigm is counterproductive. We accept that rights can be effective for advancing environmental policy goals. However, we argue, rights are neither necessary nor obviously desirable for advancing green normativity. Empirical studies support this conclusion: rights-based frameworks structure holistic ecological concerns as an interest-balancing exercise, where ecosystems compete with other legal persons for rights to use or control natural resources. Green constitutionalists should therefore consider a different paradigm.

Finally, we propose our alternative: the governance paradigm (section 5). Whereas the proliferation of the RoN model has been driven predominantly by norm entrepreneurs from the Global North,^8^ the governance paradigm takes its cue from the Indigenous-led ecological movements that underpin the most successful RoN laws,^9^ and draws attention to constitutional innovations from the Global South that provide possible alternatives to rights. Unlike the rights-based paradigm, which adopts a litigation-led perspective that makes courts the principal agents of green normativity, the governance paradigm emphasises the configuration of authority relations. This includes the institutions and structures through which political power is exercised and its manner of exercise: in other words, who speaks for ecosystems and how they speak. Instead of RoN, the governance paradigm supports *ecocentric legal frameworks*: an alternative model that prioritises duties to promote ecological well-being and modes of decision making based on ecological community membership. Concretely, as a matter of constitutional design, our proposal suggests that green constitutionalists should place less emphasis on judicially enforceable rights as vehicles for advancing green normativity and give greater attention to constitutional mechanisms designed to support norm change and resilience, including directive principles and fourth branch guarantor institutions. As a matter of legal theory, our proposal also renews interest in the Razian idea of ‘intrinsic duties’,^10^ suggesting that ecocentric duties are better understood as duties that arise from ecological community membership—not as duties based on nature’s ‘rights’.

## 2. Green Constitutionalism and the Case for Rights of Nature

This article examines the case for RoN as an aspect of green constitutionalism. That is, we are interested in the capacity of RoN to reconfigure constitutional norms in a manner that aligns with green normative theory. Our analysis draws on qualitative empirical studies of RoN laws and evaluates the current conceptual models that have emerged from the RoN literature. Before turning to these matters (section 3), we begin by situating our project within the literature. This includes scholarship on environmental constitutionalism, which is not necessarily focused on norm production or green normativity, as well as RoN scholarship, which is not necessarily focused on constitutional law.

### A. Background

#### (i) Environmental policy and constitutional law

From an environmental policy perspective, the interest in constitutional law lies in its capacity to advance environmental objectives. Constitutional law is viewed as an instrument of environmental policy rather than the object of study: as complementary and supplementary to other domestic law tools for achieving the same ends. Environmental law’s domestic toolkit was initially comprised of reactive, litigation-led strategies, such as tort law actions in nuisance or negligence. Since the 1970s, however, it has encompassed more proactive, regulatory mechanisms, such as environmental impact assessment and planning law.^11^ Nevertheless, environmentalism’s constitutional turn has been dominated by a focus on litigation-led strategies.

As constitutional social and economic rights have become more mainstream, there has been growing interest in environmental constitutional rights. Most prominently, this includes public interest litigation based on constitutional rights to a clean and healthy environment.^12^ Proponents of environmental constitutional rights emphasise the relative advantages of constitutional law over other domestic law sources: namely, its capacity to override ordinary law and bind state actors.^13^

More recently, this interest in constitutional litigation as an environmental policy tool has been extended to RoN. RoN are characterised as the next logical step in the spread of environmental constitutional rights,^14^ with Ecuador’s adoption of RoN in its 2008 Constitution provoking international interest. RoN are particularly attractive because they overcome challenges with establishing standing to bring a lawsuit,^15^ which generally requires demonstrating a particularised harm or injury to a person or a person’s property. The recognition of nature *itself* as a rights-bearing legal subject overcomes this obstacle by empowering humans to bring lawsuits on nature’s behalf when nature is harmed or injured.

#### (ii) Environmental, non-anthropocentric and green constitutionalism

Much of the RoN literature reflects this litigation-led perspective, viewing constitutional law as an instrument of environmental policy. By contrast, constitutionalism itself—that is, the phenomenon of governance according to law—is the object of our study. Specifically, we are interested in examining the promise of RoN for *green constitutionalism*, understood as a category of *non-anthropocentric, environmental constitutionalism*. It will be helpful to clarify this terminology.

The term ‘environmental constitutionalism’ is used in various ways and encompasses a wide range of projects. At its core, however, it is principally concerned with environmental governance and the institutions and legal forms that are necessary to govern with an environmental agenda.^16^ (Although our focus is on domestic law, there are also significant international law and externally facing international relations dimensions to these issues, which have been examined by other scholars.^17^) Environmental constitutionalism could be anthropocentric in orientation, reflecting a fundamental concern for human well-being, and thus broadly compatible with liberal constitutionalism. Alternatively, it could take a non-anthropocentric turn, aiming to transform the human-nature relationships associated with the dominant liberal model.

‘Green constitutionalism’, in the sense used here, is a non-anthropocentric form of environmental governance, where political power is organised to advance the commitments of what we refer to as ‘green normative theory’. We use this term to capture core features of philosophical theories of value that assign intrinsic value to nature or natural entities, cast in their most plausible (and thus most defensible) light, drawing on a range of positions defended in the literature on ecocentric environmental ethics. The central commitments of green normative theory, in the sense in which we use that term, are:

(i) *ecocentrism*, or the view that ecosystems have intrinsic value and moral standing in their own right, which in turn entails a commitment to
(ii) *the interdependency of value*, referred to as ‘holism’.^18^

Finally, green normative theory is committed to:

(iii) *ecocentric ways of valuing*, or practices that are consistent with the holism entailed by ecocentrism.

These commitments are best explained by way of contrast with liberalism and comparison with other, related, normative theories.

First, ecocentrism challenges liberalism’s anthropocentrism. Ecocentrism does not deny that nature has instrumental value for humans, but rather rejects the thesis that nature is *only* instrumentally valuable for humans.^19^ Second, holism challenges liberalism’s emphasis on individualism and the ‘separateness of persons’^20^—whether ontological (as a claim about the nature of the human self) or methodological (as a claim about how to give moral regard to humans). At the same time, this commitment also distinguishes green normative theory from other non-anthropocentric normative theories. For example, sentience-based theories do not assign intrinsic value to ecosystems and may place greater emphasis on the well-being of individuals qua individual.^21^

This point requires further clarification. Some versions of ecocentrism adopt a strong form of ontological holism that is incompatible with the moral standing of individuals (human or non-human).^22^ However, ecocentrism does not *require* adopting this position. Green normative theory (in our sense) adopts a version of ecocentrism that is compatible with moral concern for the well-being of individuals (human and non-human), who have intrinsic value as members of the ecological community.^23^ This position is analogous to strands of communitarian political thought that recognise the interdependency of value between individuals and social community, meaning that although individuals have independent value, what is good for individuals cannot be understood in isolation from what is good for the community.^24^

Green normative theory (in our sense) also places greater emphasis on methodological (as opposed to ontological) holism. This is reflected in the commitment to ecocentric ways of valuing. Whereas ontological holism insists that individuals do not exist separately from ecosystems (in some sense or other), methodological holism need not take such a position. Its focus is on promoting forms of practical reasoning that recognise the interdependency of value just described: that is, practices that treat individuals (human and non-human) as ecological community members (for example, by recognising systemic relationships).^25^ Thus, unlike strong ontological holism, methodological holism does not purport to eliminate conflicting interests by redefining the moral subject as an all-encompassing whole. It does, however, require forms of decision making that treat interests in an interdependent and systemic manner—that is, having regard to their collective contribution to ecological well-being.

In summary, if a central function of constitutions generally is to translate political power into lawful authority, then a *green constitution* translates political power into lawful authority through promoting the ends of green normative theory; namely, the well-being of ecosystems and ecological community members. This can be contrasted with a *liberal constitution*, which aims at promoting human well-being through an emphasis on the fundamental values of freedom and autonomy.^26^ Thus understood, a central problem for theorising green constitutionalism lies in understanding how an ecocentric orientation alters the organising principles, legal forms and institutions found in the more familiar liberal conception.^27^ With this challenge in mind, we turn to the *prima facie* case for RoN in a green constitution.

#### (iii) Green constitutionalism and rights of nature

Rights have a prominent place in liberal constitutions and are thought to play an important, if not essential, role in the lawful exercise of political power.^28^ It is not obvious, however, that rights play such a role in a green constitution. For example, in her pioneering work on green political theory, Eckersley questions the place of rights in a green state. She argues that ‘rights discourse … becomes considerably strained and unworkable (ontologically, politically and legally)’ if applied to aspects of the ‘biotic community’ that cannot be accommodated by the ‘individualistic premises’ that underpin rights discourse.^29^ Similarly, many Indigenous polities have been governed in accordance with green normative principles for generations before RoN came onto the scene.^30^ Moreover, those Indigenous polities that have adopted RoN have often done so with reservations, as liberal notions of ‘rights’ are foreign to their normative systems.^31^ Likewise, scholarship in critical ecology has suggested that obligations rather than rights better reflect facts about holistic social ontology.^32^

As an initial matter, then, there are reasons for scepticism about the capacity of rights-based frameworks to advance green normativity. We revisit these concerns in section 4. For present purposes, we set them aside and turn to the *prima facie* case for RoN as an essential element of green constitutionalism.

In brief, the case for RoN rests on providing a non-anthropocentric alternative to conventional environmental constitutional rights: most prominently, rights to a clean and healthy environment.^33^ These rights tend to protect the environment as an aspect of human flourishing. For instance, jurisdictions that lack express environmental constitutional rights have frequently implied them from constitutionally guaranteed human rights to life, liberty and equality.^34^ Rights to a clean and healthy environment thus do not seem very attractive from a green constitutionalist perspective. In fact, they may threaten ecocentric ways of valuing by perpetuating views of nature as a resource for humans. By contrast, RoN promise to disrupt anthropocentric liberal norms through the juridical reclassification of ecosystems as rights-bearing legal subjects—and therefore as ‘persons’, not ‘property’. In doing so, RoN recognise that ecosystems have intrinsic value, demanding moral concern in their own right.

This green normative potential has been at the forefront of RoN advocacy in recent decades. Led by the Earth jurisprudence movement,^35^ which has successfully campaigned for RoN laws in many jurisdictions throughout the world,^36^ there are increasing calls for constitutionalising RoN.^37^ Proponents characterise RoN as a ‘paradigm shift’: reorienting environmental governance away from a neoliberal model premised on resource extraction, which aims to prevent or mitigate ecological harm, and towards an ecocentric model premised on systemic interdependency, which aims to promote ecological flourishing.^38^ If advocates are correct, then RoN should have a central role in transformative green constitutionalist projects. It is this claim that we investigate.

## 3. Method: Empirically Grounded Juridical Analysis

Our analysis of existing conceptual models and our proposed alternative are based on a systematic review of the RoN literature and a meta-analysis of qualitative empirical studies of RoN laws within existing scholarship. Although representative—focusing on the most significant studies in the literature—our survey is not, of course, comprehensive. The proliferation of RoN laws has been rapid, with new proposals underway even at the time of writing,^39^ and RoN scholarship has kept pace.

This section first situates our project within the current RoN literature, explaining our research method. It then turns to the empirical foundation for our analysis (section 4) and proposal (section 5), describing the current paradigm used to model RoN laws and key examples of each of the two current models.

### A. Juridical Analysis of Constitutional Norms

RoN redeploy juridical concepts associated with liberalism (viz ‘rights’ and ‘legal personality’). However, most of the RoN literature does not critically examine these legal forms—embracing a rights-based paradigm, but without asking whether rights are necessary or desirable for advancing green normativity. In undertaking that task, we join an important body of RoN scholarship, led by O’Donnell, Macpherson and co-authors,^40^ that moves beyond the labels ‘rights’ and ‘legal personality’ and provides contextual analysis of the legal regimes established by RoN laws, including how they are institutionalised and operate in practice. We see this scholarship as analogous to an older body of qualitative empirical research on resource governance from the social sciences, following Ostrom’s classic work on common pool resources,^41^ albeit from a socio-legal tradition.

This article is intended to complement this scholarship. However, our approach differs in a few important ways. First, our project is a work of legal theory that examines the juridical dimensions of RoN. Thus, although empirically grounded, our project uses philosophical methods of analysis. In attending to the institutional and operational dimensions of RoN, we are concerned with the internal point of view of actors in a legal order, who regard themselves as bound by the conditions of legality and the specific powers, rights and duties that flow from it.^42^ Moreover, the juridical perspective that we are interested in is constitutional in character. Specifically, we are interested in the capacity of RoN to inform the understandings of actors in relation to the conditions for the lawful exercise of political power, or governance according to law.^43^ Our approach therefore differs in both its aims and methods from socio-legal scholarship, as well as from scholarship that is oriented towards predictive or inference-oriented explanations.

Second, our approach differs from the prominent legal pluralistic strand within socio-legal RoN scholarship, which emphasises Indigenous self-governance. Although RoN laws are sometimes adopted strategically to resist settler-colonial law,^44^ many Indigenous polities have used RoN laws to protect or else restore governance in accordance with established ways of living with and relating to nature, which reflect broadly green normative commitments. The legal pluralistic strand focuses on the latter trend, examining the capacity of RoN laws to create conditions for Indigenous self-governance.^45^ There is a sense in which these forms of governance can, at least arguably, be understood as a form of green constitutionalism (in our sense). Accordingly, we see this scholarship as critical for evaluating the case for RoN. Nevertheless, in drawing on insights from this scholarship, it is important to acknowledge that we have a different set of aims and methods.

### B. Current Models and Empirical Foundation

Our analysis is informed by the two conceptual models identified by Kaufmann and Martin: the *Nature’s Rights model* and the *Legal Personhood model*.^46^ Each model is an ideal type based on existing RoN laws: RoN laws can (and do) combine aspects of each. We focus on the example of each model that has proven the most influential and held up as exemplary in the literature, drawing on other examples where helpful to illustrate key points.

Despite differences in scope and juridical structure, which we describe below, both models use a *rights-based paradigm*. That is, each is presented as way of constituting nature as a rights-bearing legal subject: the Nature’s Rights model creates new rights (thereby implying that nature is a legal person qua rights bearer), whereas the Legal Personhood model creates new legal persons (thereby imbuing nature with rights-bearing capacity). This, in turn, is said to be the key mechanism through which RoN laws advance green normativity. Moreover, both are *direct models* in that they explicitly recognise nature as a rights-bearing legal subject. This can be contrasted with *indirect models*, where an entity that is uncontroversially recognised as a legal person (eg a statutory authority or corporation) is said to constitute nature as a rights bearer by creating a functionally equivalent set of jural relations.^47^

#### (i) Nature’s Rights: Ecuador (2008 Constitution)

The Nature’s Rights model most closely reflects the litigation-led focus of early attempts to create RoN laws. It confers rights on natural entities (usually, nature in general), and grants general standing to sue on nature’s behalf. RoN laws following this model typically abstain from conferring any duties to exercise this standing to promote or protect the rights in question. The Nature’s Rights model is thus reactive or reparative, in the sense that the rights conferred are only legally protected through actions for redress of immanent violations or violations that have already occurred.

The most influential example is the 2008 Constitution of Ecuador, which is the most well-known RoN^48^ and often ‘seen as the pioneer that led the way’.^49^ In fact, local government ordinances in the United States provide the earliest and most prevalent examples of the Nature’s Rights model.^50^ However, according to existing studies, these laws are designed to supplement human rights to a clean and healthy environment and thus have a broadly anthropocentric orientation, positioning human rights against corporate rights.^51^ Emerging tribal nation laws in the United States seem more promising on this front. However, at least so far, their dominant role appears to lie in negotiating intergovernmental relations (eg resisting federal law), rather than advancing green normativity^52^—although, of course, these objectives may overlap.

The Constitution of Ecuador, by contrast, reflects green normative commitments. It recognises Pachamama (an Andean Indigenous term translated as ‘Mother Earth’) as having ‘the right to integral respect for its existence and for the maintenance and regeneration of its life cycles, structure, functions and evolutionary processes’,^53^ and the ‘right to be restored’.^54^ The Constitution also recognises human rights ‘to benefit from the environment and the natural wealth’ in order ‘to enjoy the good way of living’.^55^ This notion of ‘good living’ is based on the broadly ecocentric Andean Indigenous principle ‘sumak kawsay’, which embodies ‘a holistic approach’ that places ‘emphasis on maintaining balance within natural systems’.^56^ This ecocentric understanding is reinforced by a range of state obligations to promote ecological well-being.^57^

Ecuador’s Pachamama is ‘increasingly seen as a blueprint’ for RoN laws, particularly in the constitutional context: until Mexico’s constitutional amendments in April 2024, it was the only formally entrenched or ‘capital-C’ constitutional RoN at the national level.^58^ In 2010, Bolivia adopted framework legislation conferring rights on Mother Earth,^59^ which was closely modelled after Ecuador’s Pachamama. In practice, however, the Bolivian legislation is predominantly used to assert territorial sovereignty and avoid scrutiny from the international community.^60^ Indeed, there is evidence that Bolivia’s RoN have enhanced, rather than curtailed, neoliberal economic policy by giving it greater perceived legitimacy.^61^

#### (ii) Legal Personhood: New Zealand (Te Awa Tupua Act and Te Urewera Act)

The Legal Personhood model confers legal personality on nature (usually, a specific ecosystem), and appoints a ‘guardian’ to represent nature’s interests. Guardians have legal rights to protect and promote these interests, and, at least ideally, to participate in processes of institutional decision making that are proactive and forward looking, rather than merely reactive.

The most influential examples of the Legal Personhood model are found in two New Zealand statutes: the Te Awa Tupua Act,^62^ which concerns a river, and the Te Urewera Act,^63^ which concerns a forest. Neither Act gives nature ‘unique, intrinsic rights’ as in the Ecuadorian example; instead, they grant ‘human rights associated with legal personhood’.^64^ Nevertheless, both reflect the green normativity of Māori legal traditions.^65^ For instance, the Te Awa Tupua Act describes the river as ‘an indivisible and living whole … incorporating all its physical and metaphysical elements’,^66^ and recognises Tupua te Kawa, a set of Māori beliefs about the holistic metaphysical and spiritual relationship between the river and other life, which ‘comprises the intrinsic values that represent the essence of Te Awa Tupua’.^67^ The Te Urewera Act contains similar provisions.^68^

Both Acts are constitutional in the sense that they create frameworks for decision making and restructure authority relationships.^69^ The Te Urewera Act removed the forest land from Crown governance by changing its status from national park to legal person, and restored Indigenous governance by appointing the Ngai Tūhoe iwi as the forest’s legal guardian.^70^ The reconfiguration of decision-making authority under the Te Awa Tupua Act is more complex given the multiplicity of stakeholder groups (Māori and non-Māori). New institutions were created to reflect diverse interests, which do not necessarily reflect ecocentric concerns (eg tourism interests).^71^

The New Zealand Acts are generally regarded as the most promising examples of the Legal Personhood model and have proven influential. Courts in India, Bangladesh and Colombia have ‘borrowed’ the New Zealand example as a remedy for the breach of other constitutional rights, recognising rivers as legal persons. However, human rights underpin these judicial rulings, which suggests that their green normative potential may be more limited.^72^ Moreover, the Indian and Bangladeshi decisions were based on a narrow understanding of the New Zealand Acts’ guardianship arrangements and have generally not been implemented.^73^ The Colombian example fares better on both fronts.^74^

## 4. Evaluating Rights of Nature

In asking whether nature needs rights, we are interested in investigating whether RoN should occupy a prominent place in green constitutionalist projects. This, in turn, requires evaluating whether recognising nature as a rights-bearing legal subject is accompanied by *costs* to green normativity that outweigh any corresponding *benefits* (section 4C). In answering this question, we think that it is instructive to begin with two preliminary questions: first, whether nature *can* have rights (section 4A); and second, whether rights are in any sense *necessary* (or, at least, especially beneficial) for advancing green normativity (section 4B).

Current debates about RoN in the rights theory literature, which we survey below, shed light on these questions. Even if unsuccessful, objections to RoN—which focus on the conceptual possibility of rights possession—draw attention to an aspect of rights attribution that is difficult to assimilate with green normative theory. This is, namely, the tendency of rights to reinforce a theory of value that is individualistic and atomistic, rather than holistic. This tendency is exacerbated by the paradigmatic adjudicative function of rights in constitutional practice and reflected in common features of judicial reasoning such as balancing and proportionality testing.

We ultimately conclude that green constitutionalists should avoid assigning any foundational role to RoN. In reaching this conclusion, we do not commit ourselves to any claim concerning the conceptual possibility of nature having rights. Our objection is to the likely (if not inevitable) distortionary effects of RoN as part of an expansion of liberal legal frameworks. Rights as they are invoked in our political and legal culture have certain features, and acknowledging these features suggests that green constitutionalists require an alternative, which we advance in section 5.

A point of clarification before we proceed: our discussion focuses on the attribution of rights rather than legal personhood. As noted in section 3, both current conceptual models use a rights-based paradigm: each is presented as a means of constituting nature as a rights-bearing legal subject. The rights-based paradigm thus treats rights attribution as functionally equivalent to the conferral of legal personality. This reflects the close conceptual relationship between rights possession and legal personhood in liberal theory. On the classical, or ‘orthodox’, account, someone is a legal person if (and only if) they can possess rights.^75^ Moreover, even accounts that depart from this orthodoxy view the possession of certain kinds of rights as a necessary (though not sufficient) condition of legal personhood.^76^ Therefore, to the extent that arguments against the conceptual possibility, necessity or desirability of recognising the legal personhood of natural entities succeed, they can also be deployed as arguments against recognising these entities as possessing legal rights which provide the incidents of legal personhood (and *vice versa*).

### A. Can Nature Have Rights?

Within rights theory, the debate about RoN has focused mainly on whether nature can have rights,^77^ rather than whether RoN ought to be legally recognised or protected.^78^ Arguments against RoN as conceptually proper legal rights generally purport to demonstrate their incompatibility with sound theoretical accounts of the function of rights. Theories of rights have traditionally divided into ‘will’ (or ‘choice’) theories and their rival ‘interest’ theories. Will theories emphasise rights bearers’ control over the duties of others: to have a right is to have a certain kind of control over these duties, such as powers of waiver or fulfilment.^79^ Interest theories, in their various forms, argue that a necessary function of rights is the protection of a rights bearer’s interest (or, equivalently, an aspect of the rights bearer’s well-being).^80^

The will theory is difficult to reconcile with RoN, since ecosystems are not typically thought to exercise choices or exert control over the duties of others.^81^ The debate over the possibility of RoN has thus focused on whether RoN can be accommodated within an interest theory. RoN proponents have argued (often tacitly) that nature possesses interests that are sufficient for having rights.^82^

Arguments against RoN either claim that nature does not possess ‘interests’ of the sort necessary for rights possession or that its interests lack the requisite moral status. Feinberg argues that natural entities such as trees and wilderness areas do not have rights, because they do not possess interests—they have no good of their own.^83^ While he refrains from concluding that nature cannot have rights, Raz argues that those whose well-being lacks ‘ultimate’ or non-derivative value cannot possess rights.^84^ Kurki and Kramer argue that because nature is not sentient, nature does not have the ability to have its interests harmed from its own point of view.^85^ As Kurki puts it, non-sentient natural entities cannot ‘have a stake in how things go for them’.^86^ While they have interests, they cannot be wronged, and thus cannot possess rights whose violation correlates with any wrongdoing.

For the most part, RoN proponents are unlikely to be troubled by arguments of this sort. They are certainly not troubled by the idea that nature cannot speak for or represent itself (cannot exercise its own will).^87^ Indeed, Stone’s argument for RoN in the early 1970s, usually credited with originating the RoN movement,^88^ is explicitly modelled on legal structures that protect the rights of children and legal incompetents.^89^ Nor are RoN proponents likely to share any antipathy to the idea that nature has interests, and that these interests are intrinsically valuable aside from their contribution to human well-being.^90^ Moreover, green normative theory rejects sentience as the basis for possessing intrinsic value.^91^

Indeed, RoN proponents may well regard the entire debate as a distraction. After all, RoN have already been embedded in many legal systems. As a simple descriptive matter, then, it seems obvious that nature can have rights.^92^ Moreover, even theorists who argue that nature cannot possess rights appear to concede that RoN may be capable of instrumental or symbolic justification, and that the duties that RoN create can be justified on the basis that they promote the well-being of human and non-human animals who depend on the flourishing of the relevant ecosystems.^93^ So, why take rights theorists’ objections seriously?

We think that there are at least two reasons why engaging with these arguments matters for evaluating the green normative potential of RoN. First, these arguments threaten the underlying conceptual coherence of legal RoN arrangements. Critics argue that even those rights that laws explicitly attribute to nature are not *legal rights* in the strict sense.^94^ For these critics, the most plausible understanding of RoN is that they establish a kind of legal fiction, according to which certain natural entities should be treated as if they were the bearers of legal rights or personhood.^95^

This legal fiction may well be justified for instrumental or symbolic reasons. But then it is important to investigate whether the interests protected by RoN might be more effectively protected by other legal mechanisms that directly recognise those interests. For example, if, as Kurki suggests, the true rights bearers protected by the Te Awa Tupua Act are the human and non-human animals who depend on the river,^96^ then it is worth considering the possibility that the aims of the legislation would be better pursued by recognising these rights bearers directly. In other words, if RoN are legal fictions, then this suggests that they are not, strictly speaking, necessary for advancing green normativity.

The second reason why this debate matters is that it draws attention to features of rights-based frameworks that green constitutionalists need to contend with. Arguments against RoN as proper legal rights demonstrate the conceptual burdens that need to be overcome by proponents. Even if these burdens can be overcome—we are inclined to think they can, although we do not defend this position—considerations from rights theory suggest that doing so comes at a cost to the commitments of green normative theory. Thus, to the extent that RoN have instrumental or symbolic value for advancing green normativity, then here, too, it is important to consider alternatives.

### B. Are Rights Necessary (or, At Least, Especially Beneficial) for Advancing Green Normativity?

So far, we have seen that in order to hold that nature can have rights, RoN proponents need to accept: (i) that nature can possess interests; and (ii) that these interests have intrinsic value aside from their contribution to the well-being of human and non-human animals. Green normative theory is consistent with these propositions. But is recognising nature as a rights-bearing legal subject necessary for advancing green normativity? And even if not strictly necessary, is it especially beneficial?

RoN proponents who wish to argue that RoN are essential for green constitutionalism need to demonstrate that framing the demands of green normative theory in terms of rights carries some benefit that alternative framings do not. Approaching this question on an intuitive level, it appears that the concerns that RoN laws address could be addressed without recognising nature as a rights-bearing legal subject. For example, at least formally, problems of standing could be addressed by conferring standing on a broader class of existing legal persons than have traditionally been regarded as affected by environmental harm.^97^ Moreover, the novel guardianship arrangements associated with the two New Zealand Acts could equally be achieved indirectly by the creation of legal corporations tasked with protecting these ecosystems.^98^ Similarly, the rights to existence and restoration conferred on Pachamama by the Ecuadorian Constitution could have been recast in terms of correlative public duties and liabilities. As scholars have observed, duties play an equally, if not more significant, role than rights in environmental constitutionalism generally.^99^

RoN proponents have themselves acknowledged that the conferral of rights on nature might be unnecessary to realise the aims of green normative theory. As Stone put it in his groundbreaking article, ‘one might well ask whether much of what has been written could not have been expressed without introducing the notion of trees, rivers and so forth “having rights”’.^100^ One way to frame Stone’s observation is in terms of the close conceptual relationship between rights and duties. On the prominent Hohfeldian analysis, assertions that individuals possess rights are equivalent (in some sense) to assertions that some other individual or group is under a correlative duty.^101^ Thus, the ‘right to restoration’ enshrined in the Ecuadorian Constitution, for example, correlates with a state duty to ensure that effective mechanisms for restoration are enacted.^102^ So: what, if anything, is gained by formulating such duties in terms of rights rather than simply as duties?

One possible response is that there are benefits in formulating legal duties in terms of rights possession: doing so carries with it the claim that the rights bearer’s interests are independently worthy of concern, and therefore worthy of more than indirect legal protection.^103^ For example, by conferring rights on a river, we recognise the possibility of injury to the river itself, rather than injury to the interests of those who use or benefit from the river. Treating nature as a rights-bearing entity thus seems to require abandoning anthropocentricity.

We do not deny that RoN are associated with these benefits. However, once again, it is not obvious that rights are strictly necessary in this regard. For instance, there are legal frameworks that appear to achieve the same end by recognising rivers as ‘living beings’ or ‘living entities’ instead of rights-bearing legal subjects.^104^ Thus, whatever benefits RoN might have in this regard need to be weighed against the potential downsides of rights-based normative frameworks as instruments of green normativity (which we address in section 4C, below).

Another, more promising, response is that rights play a foundational role in generating the duties owed to nature. This is implicit in Stone’s suggestion that rights bring into ‘the legal system a flexibility and open-endedness that no series of specifically stated legal rules … can capture’.^105^ This aspect of Stone’s argument lends itself to a *justificatory* account of rights.^106^ The distinction between justificatory and non-justificatory versions of the interest theory of rights introduces another, more technical, way for RoN proponents to argue that rights are necessary for advancing green normativity.

According to justificatory accounts, if an individual has a legal right, then that right is a reason for holding others to be under a duty or set of duties.^107^ On Raz’s canonical formulation of the interest theory, for example, the right must be grounded in the individual interests of the rights bearer: that is, the individual’s interests must be a sufficient reason for holding another to be under a duty.^108^ To say that an ecosystem has ‘a right to be restored’ on a justificatory account is thus to say that the ecosystem’s interests are a sufficient reason for recognising that others have a duty to restore it from harm. The important point here is that such a view supports the claim that rights are essential for advancing green normativity; on a justificatory account, rights perform a juridical role in the generation of duties that is, even if not strictly necessary, difficult to emulate.

Moreover, a natural corollary of justificatory accounts is that rights (or at least the interests that they protect) have a necessarily dynamic aspect: the same right can provide the basis for generating new duties, depending on the circumstances.^109^ Raz’s insistence that no ‘right can be exhaustively stated by stating those duties which it has already established’ echoes Stone’s insistence that the ‘open-endedness of rights’ cannot be captured by any series of specifically stated legal rules. This, too, can be seen as an advantage of adopting a justificatory account in support of RoN.

In short, the idea that rights perform a justificatory function—or, in Stone’s terms, the idea that rights are ‘open-ended’ and thus can generate novel duties and provide the impetus for norm change—suggests that rights may have a unique juridical role that makes them practically indispensable for any project committed to advancing green normativity. The trouble, however, is that to the extent that rights *do* perform this function, their invocation threatens other commitments that are more fundamental to green normative theory. This has to do with other features of justificatory accounts, which tend to distort green normative commitments by decoupling ecocentricity from holism. We consider this point next.

### C. The Costs of Rights-Based Approaches

Justificatory accounts support the claim that RoN have a foundational role in green constitutionalism by giving rights an important (if not indispensable) role in generating duties towards nature. But this comes at a cost. That cost is a commitment to individualism—the ‘separateness of persons’ and their interests—which is difficult to square with the commitments of green normative theory. Although the individualistic character of rights-based frameworks is often overstated,^110^ it is nevertheless fair to say that they are broadly individualistic.^111^ And justificatory accounts in particular tend to give them a strongly individualistic character. As Raz notes, the idea that rights are grounded in individual interests does not demonstrate that rights-based normative theories *must* be individualistic, but it does show that ‘it is not accidental that right-based theories have been and are likely to be individualistic’.^112^

At the conceptual level, these individualising tendencies are in tension with green normativity. This tension is clearest where ecocentricity is thought to entail strong ontological holism.^113^ But these tendencies also work against the more moderate, methodological holism of green normative theory (in our sense), which accepts that individuals have intrinsic value. This has to do with the commitment to ecocentric ways of valuing, which requires modes of decision making that recognise the interdependence of value within the ecological community. The trouble is that rights-based frameworks tend to work at cross purposes with this commitment: rather than facilitating the recognition of interdependency, they create an obstacle that must be overcome.

This concern is reflected in RoN scholarship criticising the ‘ecocentrism’ found within rights-based frameworks as insufficiently holistic. The criticism appears to be based on a perceived tendency of RoN to recast the green normative commitment to ecocentrism (which entails holism) as a ‘nature trumps’-type view.^114^ Doing so both denies that humans and other individuals have intrinsic value and downplays the systemic interdependency of values—or what these scholars refer to as ‘relationality’^115^—that is the hallmark of green normativity. There is, of course, a thin sense in which all rights are ‘relational’. Each right corresponds to a duty or a set of duties that others owe to the rights bearer, and the relationship between rights bearer and duty bearer is one of Hohfeld’s four ‘strictly fundamental’ legal relations.^116^ But, plainly, this is not the sort of ‘relationality’ required by green normativity: which is to say, it is not inherently *holistic*.

These conceptual difficulties should not be overstated. Aside from those that subscribe to the strongest forms of ontological holism, any normative theory needs to accommodate the possibility of conflicting interests. Our concerns relate to the systematisation of RoN—and, in particular, embedding RoN within adjudicative frameworks designed to resolve conflicting interests. This includes the consequences of judicialisation and well-established judicial methods in constitutional rights adjudication that tend to reinforce the individualising tendencies of rights-based frameworks.

At a purely conceptual level, it is not inevitable that inserting RoN into a given constitutional system comes at the expense of recognising the interdependence of value within the ecological community. (Rights associated with ecosystemic interests could be granted some sort of lexical priority over other rights, for instance.) At the same time, however, RoN proponents must contend with existing constitutional structures that protect and promote competing individual and group interests. As Kurki notes, the constitutionalisation of RoN appears to be intended to ensure that ecosystems ‘*better* “compete” with other holders of existing rights’.^117^ But constitutional systems where RoN compete for priority with other rights do not seem well-equipped for the normative transformation that proponents envisage.

The implementation of Ecuador’s constitutional RoN framework illustrates these distortionary effects. Pachamama’s rights ‘to exist and to maintain and regenerate its life cycles, structures, functions and evolutionary processes’ under chapter 7 of the Constitution (‘Rights of Nature’) exist alongside a human right (also under chapter 7) ‘to benefit from the environment and from the natural wealth that allows for their wellbeing’.^118^ As Alston observes, given Ecuador’s long-standing dependency on extractive industries, ‘the right to benefit from the environment directly reflects the underlying belief … that all should share in the revenues derived from their country’s natural wealth’.^119^ This includes the realisation of costly social and economic rights, which the Constitution also recognises.^120^

This potential for conflicting interests is not in itself problematic. However, within a green normative framework, the right to benefit from the environment (including for the purpose of realising social and economic rights) should be in some sense subsidiary to ecological well-being, acknowledging the systemic interdependency of values. Thus, even where these subsidiary interests are acknowledged, the flourishing of ‘life cycles, structures, functions and evolutionary processes’ should have priority. In other words, the framework for implementing social and economic rights must acknowledge that human rights bearers are not individuals who ‘compete’ with the interests of nature, but ecological community members. This is not about nature’s rights ‘trumping’ other social goals; however, ‘there is clearly an upper limit’ on using resource extraction to support these other goals.^121^

In practice, however, Pachamama’s rights do not operate in a manner that encompasses the subsidiary interests of other individuals and groups protected by the Constitution but exist alongside them in a competitive legal framework. Since the adoption of the 2008 Constitution, the Ecuadorian government has made significant use of the RoN framework to justify an extractive development agenda. Between 2008 and 2020, 13 of the 38 RoN applications were brought by the government in support of development, and all were successful.^122^

In more recent years, constitutional litigation has been more successful in using RoN to prevent environmental harm.^123^ Crucially, however, these more recent cases have not altered the normative structure of environmental decision making. Government-led cases reflect a desire to consolidate decision-making authority over resources, rather than a fundamental concern for ecological well-being.^124^ Moreover, the government’s only proactive step towards implementation, creating new criminal sanctions for breaching RoN, is predominantly used to enforce government control over mining activities.^125^ Civil society-led cases have been most successful when ‘nature’s interests’ are aligned with anthropocentric constitutional rights, including rights to private property, a clean and healthy environment, and water.^126^ In many of these cases, RoN could be (or were) used interchangeably with anthropocentric rights.^127^ Thus, although the outcomes of this litigation are environmentally desirable, they have come at the cost of entrenching a normative framework that assigns priority over competing claims for resources.

Overall, then, what Ecuador’s experience highlights is the tendency of rights-based frameworks to frame the green normative concern for ecological well-being in terms of ‘nature’s interests’, which must then be ‘balanced’ against competing interests.^128^ The Constitutional Court has recognised that RoN are ‘transversal’, which at least in principle affirms a holistic understanding of value.^129^ In practice, however, ‘The concept of nature’ functions as a lens ‘through which different communities’ interests and rights are weighed against each other’.^130^

Commonplace features of constitutional rights adjudication reinforce these individualising tendencies. Courts generally evaluate constitutional rights claims in terms of justifying state action against competing priorities. This occurs via proportionality testing or other, more flexible, methods of balancing, which are designed to ‘weigh’ interests protected by the right against the interests advanced by government policy or legislation.^131^ These techniques are poorly equipped to capture the holism of green normativity, both reducing ecological well-being to ‘nature’s interests’ and framing nature’s interests as in competition with (rather than constitutive of) the public interest. Moreover, courts generally afford a degree of deference to state-defined objectives, and this is particularly strong where the government policy or legislation gives effect to another constitutionally guaranteed interest.^132^ This makes rights that protect ecocentric concerns extremely vulnerable in the absence of implementing legislation or institutions (as the Ecuadorian experience also illustrates).^133^

These features of constitutional rights practice may help explain why, in drafting the 2008 Constitution, the coalition led by then-President Rafael Correa agreed to RoN but rejected entrenching mechanisms designed to transform ecological decision-making authority. Most notably, Correa rejected the consent requirement sought by Indigenous advocates.^134^ At the same time, the new Constitution included structural changes designed to consolidate executive power, including expanded powers over natural resources and economic development.^135^ Dixon and Landau cite Ecuador’s RoN as an example of ‘abusive constitutionalism’, where rights are used to advance authoritarian political agendas,^136^ sounding a further note of caution about assuming that constitutionalising RoN will advance green normativity.^137^ Experience in Bolivia, which has framework laws modelled after Ecuador’s RoN, also confirms this.^138^

In summary, efforts to recognise nature as a rights-bearing legal subject are often an improvement over the status quo and, in at least some cases, have proven instrumentally valuable in advancing environmental policy goals, particularly where they help overcome standing requirements. Nevertheless, these improvements may not ultimately advance green normativity—or, at the very least, they come at a cost. As such, it is incumbent upon green constitutionalists to consider alternatives.

## 5. Towards an Alternative Conceptual Model: The Governance Paradigm and Ecocentric Legal Frameworks

So far, we have considered challenges that face the rights-based paradigm, which holds that the key mechanism for advancing green normativity lies in recognising nature as a rights-bearing legal subject—whether through conferring rights (the Nature’s Rights model) or legal personality (the Legal Personhood model). The purpose of a conceptual model is to provide a representation of an empirical phenomenon that assists in understanding and evaluating that phenomenon in light of some objective, delineating its basic principles and functionality. Thus, in order to serve the transformative objective of green constitutionalism, a sound conceptual model of the legal frameworks studied in RoN scholarship needs to provide the basis for understanding and evaluating how these laws advance green norms, capturing both what makes them successful and how they can be improved.

The rights-based paradigm and the models that it has produced are not fit for this purpose. On the one hand, rights are not strictly necessary: the symbolic and instrumental benefits associated with recognising nature as a rights-bearing legal subject could be achieved through alternative legal frameworks. On the other hand, to the extent that rights do have a justificatory role that is difficult to emulate with alternative legal frameworks, they come at a cost: rights are associated with a form of individualism that is problematic within green normative theory, and justificatory conceptions particularly so.

In our view, the rights-based paradigm errs in relying on a litigation-led perspective and placing insufficient emphasis on decision-making authority. Rights adjudication is treated as the primary mechanism for advancing green normativity. Although this may help advance environmental policy goals, we do not think that it is particularly beneficial for the green constitutionalist project, which seeks to transform the human–nature relationships embedded in constitutional norms. By adding ecosystems to the population of rights-bearing legal subjects, the rights-based paradigm leaves the natural world divided up into discrete individuals competing for power over resources and risks further entrenching anthropocentric liberal norms.

Green constitutionalism needs a new conceptual model. In this section, we propose an alternative approach to modeling: the governance paradigm. Whereas the rights-based paradigm emphasises protecting nature’s ‘interests’, our alternative emphasises authority relations. Instead of repurposing liberal legal forms, we start with the commitments of green normative theory and ask, ‘what legal mechanisms seem best suited to promote decision making that reflects ecocentric values and ways of valuing?’

This approach suggests that green normativity would be better served by *ecocentric legal frameworks*. Whereas RoN add new rights-bearing legal subjects to the existing community of legal persons, this conceptual model seeks to reconfigure law’s ‘persons’ as ecological community members. Concretely, ecocentric legal frameworks:

(i) *recognise ecocentric values*, assigning intrinsic value to ecosystems as holistic ‘living beings’ (rather than ‘legal persons’ with protected interests);
(ii) *emphasise ecocentric duties*, or duties to promote ecological well-being that reflect holism (ie the systemic interdependency of values), which are a type of ‘intrinsic duty’ and *not* derived from or justified by rights; and
(iii) *institutionalise ecocentric decision making*, creating institutions and structures that support governance in accordance with these values and duties.

As a matter of constitutional design, and without being prescriptive, we suggest that innovations from the Global South designed to support norm change and resilience and that provide alternatives to rights—including, although not limited to, directive principles and guarantor institutions—merit greater consideration.

Our argument here, as with our analysis in section 4, is informed by empirical studies of the practical implementation of RoN laws. In particular, we draw on insights from socio-legal scholarship emphasising the Indigenous-led ecological movements that underpin the most successful legal frameworks. As we argue below, we think that these studies show why our proposed alternative provides a more descriptively accurate *and* more analytically powerful conceptual model (section 5A). In further support of our proposal, we also draw on Raz’s arguments about the primacy of duties in the context of certain kinds of intrinsic and collective goods, of which we think ecological well-being is representative (section 5B).^139^ Although beyond the scope of this article, we think that a renewed interest in duties of this kind holds wider significance for constitutional theory and also recommends our proposal.

### A. Ecocentric Governance through Ecocentric Legal Frameworks

Empirical studies suggest that the two New Zealand Acts have had the most success to date in advancing green normativity, whereas Ecuador’s Pachamama has had much more limited success, despite substantial constitutional RoN litigation. The governance paradigm captures the key features that have made the New Zealand Acts more successful. It also captures why the Pachamama framework has fallen short, indicating how the framework could be improved. By contrast, the rights-based paradigm is not well-equipped to explain this. Indeed, it presents the relative success of the New Zealand Acts as counterintuitive or paradoxical because it associates the strength of legal frameworks as instruments of green normativity with providing adjudicative mechanisms for norm production.^140^

Overall, the rights-based paradigm tends towards a reductive view of the New Zealand Acts on two fronts. First, it encourages fixation on ‘legal personhood’. However, the attribution of legal personality (and its corollary, the capacity to bear rights) is neither necessary nor instrumental to how these regimes advance green norms: it was merely a device to circumvent questions of ‘ownership’ and manage human relationships.^141^ Both Acts place greater emphasis on legal recognition of the ecosystem as a living and holistic entity—a ‘living being’ rather than a ‘legal person’^142^—and ecological community membership. For example, decision-making principles under the Te Awa Tupua Act ‘emphasise the living, integrated and holistic nature of the river’ and the ‘responsibility of everyone with interests in the river to work collaboratively to further the river’s health and well-being’.^143^ Similarly, recent ecocentric legal frameworks adopted in Australia do not rely on rights or legal personality at all, in part because they are thought to distort the broadly green normative commitments of the Indigenous legal principles that inform them.^144^

Contra the rights-based paradigm, the mechanism for advancing green normativity thus does not lie in recognising an ecosystem as a rights-bearing legal subject, or by prioritising the ecosystem’s interests in natural resources (trees in the case of Te Urewera, or water in the case of Te Awa Tupua) over the competing interests of other rights bearers. Instead, consistent with the governance paradigm, the mechanism lies in establishing decision-making institutions designed to act in accordance with Māori normative principles, te Kawa o Te Urewera and Tupua te Kawa, which reflect the commitments of green normative theory.^145^ The decisions of these authorities are not conventional land-use decisions, but rather decisions about ecological community governance: although they allocate entitlements to use resources, they do so in a manner that recognises holistic interdependency. For example, the Te Urewera framework utilises ‘friendship agreements’ to grant licences and permits to use resources, recognising the forest’s status as a living entity and humans as ecological community members.^146^ Notably, however, these entitlements are not allocated to the ecosystems themselves—a shortcoming that arguably reflects the limitations of relying on legal personality as a mechanism.^147^

By contrast, the Ecuadorian framework’s emphasis on standing to sue, modelled after Stone’s original proposal, distorts green normative commitments.^148^ Concern for ecological well-being, based on Andean Indigenous principles, is recast as concern to protect the adversarial interests of Pachamama, who requires guardians because she is weak. Moreover, it directs attention away from the duty-based institutional and structural mechanisms needed to effect norm change and resilience: anyone *can* sue on Pachamama’s behalf, but no one has *an obligation* to do so, and, importantly, they need not be motivated by ecocentric concerns. As discussed above, this makes ecological well-being doubly vulnerable to competing government priorities. Notably, recent Indigenous-led litigation has focused on the constitutional right to consultation rather than RoN.^149^ In practice, however, courts have treated this right as satisfied where the government provides information about decisions it has made.^150^ This falls far short of the consent requirement that Indigenous activists demanded, which would have altered ecological decision-making authority by entrenching a structural requirement.

This leads to a second problem with the rights-based paradigm, which is that it tends to distort the character of ‘guardianship’ under the two New Zealand Acts, treating it as a form of litigation guardianship.^151^ For instance, the Indian judicial decision invoking the Te Awa Tupua example—by way of constitutional ‘borrowing’—and granting legal personhood to the Ganges and Yamuna rivers relied on the *in loco parentis* doctrine.^152^ The State of Uttarakhand was appointed guardian, but no institutions or requirements for ecocentric decision making were created.^153^ The state appealed the decision, arguing that it could be legally liable for harms the rivers inflict on humans (for example, flooding their land).^154^ Although this may sound far-fetched, the findings of O’Donnell and co-authors indicate that ‘can we sue?’ is the most common response to proposals for granting legal personhood to ecosystems.^155^

The emphasis that the governance paradigm’s alternative model, ecocentric legal frameworks, places on ecocentric values, duties and decision making better captures the notion of ‘guardianship’ in play. In fact, as used in the New Zealand Acts, this term is a translation of the Māori word ‘kaitaki’, which is premised on holistic interdependency and not on nature’s weakness.^156^ ‘Guardianship’ thus has less to do with ‘acting on behalf of’ and more to do with ‘acting with’^157^ or ‘caring for’.^158^ Practically speaking, this is realised through creating institutions with duties to govern in accordance with ecocentric values. Human stakeholders are positioned as ecological community members, and decisions are made for the well-being of that ecological community—described by the Te Urewera management plan as ‘managing people for the benefit of the land’.^159^ Thus, for example, decision making under the Te Awa Tupua regime focuses on how projects ‘can promote … *abundance* in the river’, ‘encouraging decisions that obligat[e] caring for, or giving to the river’.^160^ Decision makers participate in workshops ‘to better understand and be socialised into the Tupua te Kawa values and ways of relating to the river’.^161^ This approach to ecological decision making stands in contrast to rights-based frameworks, which balance the ecosystem’s interests against the competing interests of other legal persons and emphasise redressing harm.

Moreover, the governance paradigm helps capture the relatively greater success of the Colombian Constitutional Court’s *Atrato River* ruling as an instance of constitutional ‘borrowing’ from the Te Awa Tupua example.^162^ A striking feature is the institutional character of the judicial remedy: ordering the creation of decision-making bodies with duties to promote ecological well-being.^163^ Indeed, studies suggest that recognising the river as a rights-bearing legal subject was both unnecessary and largely symbolic,^164^ and acknowledge that this symbolism comes at a cost.^165^ Similarly, the governance paradigm also draws attention to the institutional character of the remedies that feature in the most promising Ecuadorian Constitutional Court rulings in recent years.^166^ For example, in the *Los Cedros case*,^167^ which has been celebrated by RoN proponents, the Court ordered a remedy that ‘mandates a new governing structure for forest management which requires management plans and decision making’ in accordance with principles that reflect the commitments of green normativity.^168^

To be sure, neither Colombia nor Ecuador has been as successful as New Zealand in implementing these court-ordered ecocentric legal frameworks. However, many of these obstacles turn on social and political factors that make norm change and resilience more difficult in the Global South, such as political instability, violence and poverty.^169^ But it also points to the general limitations of judicially crafted remedies in rights-based constitutional frameworks, where we have seen that green normative commitments are particularly vulnerable. The governance paradigm suggests that entrenching (formally or otherwise^170^) these institutional and structural mechanisms for ecological decision making would be more effective than a rights-based framework, which leaves them to be developed (if at all) by courts and treats them as remedial responses to frustrated interests rather than a constitutional mandate that forms a requirement of lawful governance.

Concretely, we suggest that green constitutionalists would profit from looking to Global South jurisdictions, which have greater experience with transformative constitutional projects. Leaving aside distortionary concerns, experience in the Global South suggests that rights are an indirect and less reliable vehicle for norm change and resilience than constitutionally mandated institutions tasked with fulfilling duties to nourish and protect fundamental norms. Although detailed consideration of matters concerning constitutional design is beyond the scope of this article, we suggest that two important innovations that merit the attention of green constitutionalists in considering how to constitutionalise ecocentric legal frameworks are directive principles and guarantor institutions.^171^

Directive principles are obligations directed to the political branches of the state that are generally incapable of direct judicial enforcement: requirements to give effect to fundamental values rather than judicially protected interests.^172^ As an alternative to rights, directive principles thus emphasise institutionalisation over adjudication as the primary mechanism for norm change, providing ‘thick moral commitments’.^173^

Guarantor institutions support directive principles and the constitutional values that underpin them. According to Khaitan, the function of guarantors ‘is to provide a credible and enduring guarantee to a specific non-self-enforcing constitutional norm’^174^—that is, a norm that powerful actors are likely to have reason and capacity to frustrate. Guarantors are similar to regulatory bodies, but ‘doubly constitutionalised’: they are constitutional institutions, ‘entrenched’ in the sense that they cannot be easily changed by the government of the day, and they give effect to constitutional norms.^175^ As Khaitan observes, constitutional norms generally require ‘constant nourishment by further specification and interpretation’.^176^ However, in the case of non-self-enforcing norms, guarantors are better equipped than courts to perform this role,^177^ especially where the norm is aspirational or transformative.^178^

This description fits green norms. Guarantors thus seem apt for the transformative aims of green constitutionalist projects and provide an alternative to rights-based frameworks as a means of constitutionalising ecocentric legal frameworks. As Samararatne argues in her work on Sri Lanka, guarantors can achieve incremental norm change that would likely not be possible via adjudicative mechanisms—even in highly unfavourable circumstances of norm-contestation and threats from external actors, which she describes as ‘resilience by stealth’.^179^ Moreover, whereas courts must balance competing interests and afford a degree of deference to the political branches, because guarantors are ‘dedicated to protecting a single, specific norm’ they ‘are likely to give its protection enormous, and frequently decisive, weight’.^180^

In summary, the rights-based paradigm encourages a litigation-led perspective where the primary mechanism for advancing green norms is through judicial rulings that give priority to an ecosystem’s interests over the competing interests of other legal persons. Within this paradigm, the two New Zealand Acts have been described as ‘weak’ because the norms are not judicially enforced and untested in court, and because the Acts are not explicit about ‘the hierarchy of rights’.^181^ Under the governance paradigm, by contrast, the alleged ‘weakness’ of the Acts turns out to be a strength. Reconceived as ecocentric legal frameworks, the Acts reconfigure authority relations by: (i) recognising ecocentric values; (ii) emphasising ecocentric duties; and (iii) institutionalising these values and duties by creating institutions and structures that support ecocentric decision making.

Importantly, we now want to argue, the ecocentric duties that underpin decision making under the governance paradigm are a different kind of duty than duties that are derived from or justified by rights. Thus, although ecocentric legal frameworks are not necessarily incompatible with rights or legal personality—indeed, both play a role in the two New Zealand Acts—they do not *require* rights.

### B. Ecocentric Duties as Intrinsic Duties

Our preferred approach and model do not require abandoning the view that ecosystems have intrinsic value. Nor do they require abandoning the view that we have fundamental duties towards or concerning ecosystems. However, one possible objection is that generating these duties requires rights attribution. Thus, RoN proponents might argue, recognition of nature as a rights-bearing legal subject must continue to play an essential role. We think that this objection misconceives the character of ecocentric duties. On our view, the governance paradigm and the ecocentric legal framework model involve recognition of our ecological duties as ‘intrinsic duties’ towards the ecological community.^182^ These duties are not generated by rights.

An intrinsic duty, as Raz defines it, is a duty that ‘requires non-instrumental justification’, such that ‘their existence is of intrinsic, even if not necessarily of ultimate value’.^183^ A crucial feature of intrinsic duties is that they cannot be derived from or justified by rights.^184^ Raz illustrates the idea of an intrinsic duty with two examples: our duty to preserve a Van Gogh painting and our duty to compensate friends for harm that we have caused to them (even where that harm was not caused by a fault of ours).^185^ In each case, the duty itself has value, and is not justified by appeal to the interests of an individual. Our duties towards works of art form part of our duties of respect towards ‘the values which give meaning to human life’.^186^ Our duties towards our friends form part of the good of friendship itself—they are not justified by the interests of our friends but by the intrinsic value of friendship. The relationship between the value of friendship and the duties that it supports, moreover, exhibits a kind of holism that is familiar to green normative theory.

We think that a similar concern for ecocentric duties as duties possessing intrinsic value is central to green normative theory, and thus a more appropriate foundation for green constitutionalism. A key commitment of green normative theory is the idea that we have basic duties towards our natural environment that resist instrumental justification. Consider, by way of illustration, Richard Sylvan’s famous ‘last man’ thought experiment, in which ‘the last man (or person) surviving the collapse of the world system lays about him, eliminating, as far as he can, every living thing, animal or plant’.^187^ The purpose of a green normative ethic is to explain why the last man’s conduct is ‘to a greater or lesser extent evil, and hence in serious cases morally impermissible’.^188^ As Sylvan notes, the development of such an ethic does not require the attribution of rights to nature—‘persons can relate morally, through obligations, prohibitions and so forth, to practically anything at all’.^189^ The idea motivating Sylvan’s thought experiment thus seems to be that there is some intrinsic duty that is breached by the last man—a duty that arises from ecological community membership.

As with rights-based approaches, a focus on ecocentric duties as intrinsic duties carries with it the instrumental and symbolic values associated with recognising and responding to nature as having moral concern in its own right, apart from serving human well-being. Unlike rights-based approaches, however, the governance paradigm encourages the conceptualisation of ecocentric duties as intrinsic duties that form part of our relationship with the environment, rather than duties justified by the interests of isolated natural entities.

Our duties towards nature arise from our recognition of and respect for the intrinsic value of ecological relationships. For example, the green normative promise of the Te Awa Tupua regime lies partly in enabling ecological community members to act in accordance with the duties that are intrinsic to their relationship to the river. As discussed above, the Act is structured to encourage decisions that are in conformity with duties of care for the river and that promote ecological well-being.^190^ These duties are fundamental, and form part of human relationships to the river ecosystem. Similarly, we note that for all of the emphasis on Pachamama’s rights, comparatively little attention has been given to the robust duties towards nature under the Ecuadorian Constitution.^191^ It is possible that at least some of these duties may be more profitably understood not as derived from or justified by Pachamama’s rights, but as an independent source of duties in recognition of the intrinsic value of the ecological community—analogous to directive principles.

Finally, we think that our preferred understanding of ecological duties as intrinsic duties suggests that other constitutionally protected relationships—including ‘third generation’ cultural rights—may in fact be better understood as intrinsic duties, and thus better protected by duties-based legal frameworks than by rights-based legal frameworks. Although exploring this possibility is beyond the scope of this article, this strikes us as a reason why our analysis and proposal may hold wider interest for constitutional theory beyond debates about constitutionalising RoN—particularly in other areas where the individualising tendencies of rights-based frameworks are sometimes thought to distort the values and norms in play.^192^

Of course, we acknowledge that some theorists, particularly those who are steadfastly committed to Hohfeldian accounts of rights, will be inclined to reject the idea that there are intrinsic duties that do not correlate with rights.^193^ Although we think that this view is implausible, we are willing to concede that it might be possible to identify different putative rights that correlate with our intrinsic duties towards nature.^194^ Our argument is that such rights cannot be viewed as justifying the duties in question: they are justificatorily inert, and therefore provide an improper foundation for a green constitutionalist project.

## 6. Conclusion

In conclusion, we disagree with RoN proponents who argue that recognising nature as a rights-bearing legal subject is essential for the transformative aims of green constitutionalism. The rights-based paradigm has produced conceptual models that perpetuate ways of valuing and relating to nature that reflect liberal normative commitments. They characterise ecological decision making as a competition for priority between conflicting interests in the use and control of natural resources. At the same time, the concrete institutions and practices embedded in at least some RoN laws have proven to hold promise for advancing green normativity. However, the rights-based paradigm does not capture the reasons *why* they hold this promise. Our proposed alternative—the governance paradigm and the ecocentric legal framework model—is better equipped to explain this and to help green constitutionalists evaluate possible legal mechanisms for norm change and resilience.

Our analysis also demonstrates the value of jurisprudential scholarship for producing conceptual models of empirical legal phenomena. Legal theory requires an empirical base. But equally, empirical studies of legal phenomena require legal theory.^195^ The qualitative empirical studies surveyed in this article support our critique of the rights-based paradigm, and many RoN scholars have voiced reservations. And yet, the Earth jurisprudence movement and other advocates of a green normative agenda continue to focus efforts on adopting RoN—arguing that recognising nature as a rights-bearing legal subject is the most promising mechanism for advancing green normativity—with proposals to adopt constitutional RoN gaining momentum in recent years.^196^ Poor conceptual modelling, with inadequate attention to the juridical dimensions of RoN, thus appears to have prevented some RoN scholars from seeing what their own empirical studies demonstrate: namely, that nature needs green governance, not rights.

## Footnotes

1 CM Kauffman and PL Martin, *The Politics of Rights of Nature: Strategies for Building a More Sustainable Future* (MIT Press 2021) 18.

2 ibid 8.

3 See CM Korsgaard, ‘Two Distinctions in Goodness’ (1983) 92(2) Philosophical Review 169; see also K Green, ‘Two Distinctions in Environmental Goodness’ (1996) 5(1) Environmental Values 31. This is the sense in which we use the term ‘intrinsic value’ throughout the paper, unless indicated otherwise.

4 We discuss green normative theory in more detail in section 2.

5 J Raz, *The Morality of Freedom* (OUP 1988) 193–216.

6 See Kauffman and Martin, *Politics* (n 1) ch 2.

7 See A Stone and LK Weis, ‘Constitutional Theory in a Comparative Context’ in G Jacobsohn and M Schor (eds), *Comparative Constitutional Theory* (2nd edn, Edward Elgar, forthcoming) 4 <https://papers.ssrn.com/sol3/papers.cfm?abstract_id=4851469> accessed 1 April 2025; D Samararatne, A Stone and LK Weis, ‘Editorial’ (2023) 1(2) Comparative Constitutional Studies 169.

8 Kauffman and Martin, *Politics* (n 1) 52–3. The United States and Canada account for around 40% of RoN actors, followed by Europe and Latin America (each around 20.5%); Asia and Africa are both heavily underrepresented.

9 See E O’Donnell and others, ‘Stop Burying the Lede: The Essential Role of Indigenous Law(s) in Creating Rights of Nature’ (2020) 9(3) TEL 403.

10 Raz, *Morality of Freedom* (n 5) 193–216.

11 See C Wood, *Environmental Impact Assessment: A Comparative Review* (2nd edn, Routledge 2014).

12 See eg DR Boyd, *The Environmental Rights Revolution: A Global Study of Constitutions, Human Rights, and the Environment* (UBC Press 2012); T Hayward, *Environmental Constitutional Rights* (OUP 2004).

13 See LK Weis, ‘Environmental constitutionalism: Aspiration or transformation?’ (2018) 16 ICON 836, 839–42.

14 See J Gilbert, ‘Human Rights & the Rights of Nature: Friends or Foes’ Fordham Int’l LJ (forthcoming) (manuscript also on file with the authors). RoN are usually traced to Christopher Stone’s influential article, ‘Should Trees Have Standing?—Towards Legal Rights for Natural Objects’ (1972) 45 S Cal L Rev 450. Their legal enactment is recent, however: see E O’Donnell, *Legal Rights for Rivers: Competition, Collaboration and Water Governance* (Routledge 2019) 22–3.

15 See DR Boyd, *The Rights of Nature: A Legal Revolution That Could Save the World* (ECW Press, 2017) 102–8; Stone (n 14) 459–64.

16 For a helpful survey of different approaches, see S Bookman, ‘Demystifying Environmental Constitutionalism’ (2024) 54 Environmental Law 1.

17 See eg R Eckersley, *The Green State: Rethinking Democracy and Sovereignty* (MIT Press 2004).

18 See A Thompson, ‘Anthropocentrism: Humanity as Peril and Promise’ in SM Gardiner and A Thompson (eds), *The Oxford Handbook of Environmental Ethics* (OUP 2017) 80–1.

19 Some RoN scholars criticise ecocentrism on the basis that the attribution of intrinsic value is incompatible with instrumental value and entails that nothing else has intrinsic value: see eg E Macpherson and FC Ospina, ‘The Pluralism of River Rights in Aotearoa, New Zealand and Colombia’ (2018) 25(6) Water Law 283, 285, 287, 291; M Tănăsescu, *Understanding the Rights of Nature: A Critical Introduction* (Transcript 2022) 57, 101, 132; M Tănăsescu, ‘Rights of Nature, Legal Personality, and Indigenous Philosophies’ (2020) 9(3) TEL 429, 432. This criticism appears to rest on philosophical confusion, perhaps perpetuated by rhetoric from some RoN proponents suggesting that ‘ecocentrism’ means ‘nature trumps’, or else by assuming (mistakenly) that ecocentrism entails strong ontological holism, such that there are no individuals and only one moral subject (‘Nature’).

20 See J Rawls, *A Theory of Justice* (Harvard UP 1971) 26–7.

21 See eg R Fasel and J Adenitire, *Animals and the Constitution: Towards Sentience-Based Constitutionalism* (CUP forthcoming) (manuscript also on file with the authors).

22 This is how Aldo Leopold’s ‘land ethic’, usually credited as the origin of ecocentrism, is typically understood: see D Jamieson, *Ethics and the Environment: An Introduction* (CUP 2008) 150; Thompson (n 18) 81.

23 This includes views drawing on Callicott’s Humean reinterpretation of Leopold (emphasising moral sentiments), as well as views drawing on Rolston’s objectivist environmental holism (emphasising life-producing forces): see Jamieson (n 22) 149–53; J Kawall, ‘Environmental Ethics’ in Gardiner and Thompson (n 18) 17–18; Thompson (n 18) 80–1.

24 See eg C Taylor, *Philosophical Papers*, vol 2: *Philosophy and the Human Sciences* (CUP 1985) 187; C Taylor, *Sources of the Self: The Making of the Modern Identity* (CUP 1989).

25 This reflects the turn towards pragmatism in environmental ethics, which emphasises valuing and valuational structures in an effort to bypass ontological debates between biocentrists and ecocentrists about the moral status of individuals: see Jamieson (n 22) 153–5; Kawall (n 23) 19–20.

26 See NW Barber, *The Principles of Constitutionalism* (OUP 2018).

27 cf S Tierney, *The Federal Contract: A Constitutional Theory of Federalism* (OUP 2022) (arguing that a federal constitution has a different conception of its ‘people’ and their jural relations than a unitary constitution).

28 See eg R Gargarella, *Latin American Constitutionalism, 1810–2010: The Engine Room of the Constitution* (OUP 2013) 14–17.

29 R Eckersley, ‘Liberal Democracy and the Rights of Nature: The Struggle for Inclusion’ (1995) 4(4) Environmental Politics 169, 169; see also 190–1, 193; Raz, *Morality of Freedom* (n 5) 198–203, 216 (on rights and individualism).

30 See O’Donnell and others (n 9).

31 See Kauffman and Martin, *Politics* (n 1) 73, 146–7; Tănăsescu, ‘Rights of Nature’ (n 19).

32 See eg K Birrell and D Matthews, ‘Re‑storying Laws for the Anthropocene: Rights, Obligations and an Ethics of Encounter’ (2020) 31 Law & Critique 275.

33 See LJ Kotze and PV Calzadilla, ‘Somewhere between Rhetoric and Reality: Environmental Constitutionalism and the Rights of Nature in Ecuador’ (2017) 6 TEL 401, 405–11.

34 Examples include India and Sri Lanka. For India, see *MK Ranjitsinh and Others v Union of India* [2024] INCA 280, *MC Mehta v Kamal Nath* (2000) 6 SCC 213, *Virender Gaur v State of Haryana* (1995) 2 SCC 577. For Sri Lanka, see *Kariyawasam v Central Environmental Authority* [2019] SC FR No 141/2015 (4 April 2019), *Environmental Foundation Ltd v Mahawali Authority* [2010] 1 Sri LR 1 (17 June 2010), *Watte Gedera Wijebanda v Conservator General of Forests* [2009] 1 Sri LR 337 (5 April 2007), *Bulankulama v Ministry of Industrial Development (Eppawela case)* [2000] 3 Sri LR 243 (2 June 2000).

35 See generally C Cullinan, ‘Earth Jurisprudence’ in L Rajamani and J Peel (eds), *The Oxford Handbook of International Environmental Law* (2nd edn, OUP, 2021) 237–9.

36 See Kauffman and Martin, *Politics* (n 1) 4–10.

37 See eg L Collins, *The Ecological Constitution: Reframing Environmental Law* (Routledge 2021) ch 5.

38 ibid 7; see also Kotze and Calzadilla (n 33) 411.

39 See Eco Jurisprudence Monitor, ‘Toolkit’ <https://ecojurisprudence.org/initiatives/> accessed 1 April 2025.

40 See eg L Cano-Pecharroman and E O’Donnell, ‘Relational Representation: Speaking with and not about Nature’ (2024) 3(10) PLOS Water; M Cribb, E Macpherson and A Borchgrevink, ‘Beyond Legal Personhood for the Whanganui River: Collaboration and Pluralism in Implementing the Te Awa Tupua Act’ International Journal of Human Rights (publ online 16 February 2024) < https://doi.org/10.1080/13642987.2024.2314532> accessed 1 April 2025; E Macpherson, *Indigenous Water Rights in Law and Regulation: Lessons from Comparative Experience* (CUP 2019); Macpherson and Ospina (n 19); O’Donnell, *Legal Rights* (n 14); E O’Donnell, ‘Rivers as Living Beings: Rights in Law, but No Rights to Water?’ (2020) 29 GLR 643; O’Donnell and others (n 9).

41 E Ostrom, *Governing the Commons: The Evolution of Institutions for Collective Action* (CUP 1990); E Schlager and E Ostrom, ‘Property-Rights Regimes and Natural Resources: A Conceptual Analysis’ (1992) Land Economics 249.

42 See D Dyzenhaus and T Poole, ‘The old Commonwealth model of constitutionalism’ (2023) 1(2) Comparative Constitutional Studies 197.

43 This may be understood as an exercise in ‘small-c’ constitutional analysis in that it does not turn on whether RoN are formally entrenched.

44 See Kauffman and Martin, *Politics* (n 1) 176–84, 217–19.

45 See eg Cribb, Macpherson and Borchgrevink (n 40); O’Donnell and others (n 9); Tănăsescu, ‘Rights of Nature’ (n 19).

46 Kauffman and Martin, *Politics* (n 1) 14–18.

47 This occurs (roughly) where the relevant legal person exists for the purpose of protecting nature’s interests (eg purchasing environmental water to promote the health of an aquatic system): see O’Donnell, *Legal Rights* (n 14) 38, 45, 46, 49–53.

48 See CM Kauffman and PL Martin, ‘Can Rights of Nature Make Development More Sustainable? Why Some Ecuadorian Lawsuits Succeed and Others Fail’ (2017) 92 World Development 130, 131; CM Kauffman and PL Martin, ‘How Ecuador’s Courts Are Giving Form and Force to Rights of Nature Norms’ (2023) 12(2) TEL 366, 375; Kotze and Calzadilla (n 33); M Tănăsescu and others, ‘Rights of Nature and Rivers in Ecuador’s Constitutional Court’ IJHR (publ online 7 February 2024) <https://doi.org/10.1080/13642987.2024.2314536> 1.

49 Tănăsescu, ‘Rights of Nature’ (n 19) 430.

50 See Boyd, *Rights of Nature* (n 15) 109–30; Kauffman and Martin, *Politics* (n 1) 68–70.

51 See Boyd, *Rights of Nature* (n 15); Kauffman and Martin, *Politics* (n 1) 63, 69.

52 See S Bookman, ‘The Puzzling Persistence of Nature’s Rights’ Utah L Rev (forthcoming) <https://papers.ssrn.com/sol3/papers.cfm?abstract_id=4996393>; Kauffman and Martin ibid 163, 176–84; O’Donnell and others (n 9) 415–16.

53 Constitution of Ecuador, s 71.

54 ibid s 72.

55 ibid s 74.

56 Kauffman and Martin, *Politics* (n 1) 61.

57 Constitution of Ecuador, ss 71, 72, 73.

58 Constitution of Mexico, arts 5 and 18. Other jurisdictions have proposed amendments that have yet to be approved: see Eco Jurisprudence Monitor (n 39).

59 Law No 071 of 21 December 2010 Mother Earth Rights Law (Ley de Derechos de la Madre Tierra); Law No 300 of 15 October 2012, Framework Law of Mother Earth and Integral Development for Living Well (Ley Marco de la Madre Tierra y Desarrollo Integral para Vivir Bien).

60 Kauffman and Martin, *Politics* (n 1) 119, 123–4.

61 See L Muñoz, ‘Bolivia’s Mother Earth Laws: Is the Ecocentric Legislation Misleading?’ (*ReVista*, 6 February 2023).

62 Te Awa Tupua (Whanganui River Claims Settlement) Act 2017.

63 Te Urewera Act 2014.

64 Kauffman and Martin, *Politics* (n 1) 64; see Te Urewera Act, s 11; Te Awa Tupua Act, s 14.

65 Tănăsescu, ‘Rights of Nature’ (n 19) 447, 451; Macpherson and Ospina (n 19) 284–5; O’Donnell and others (n 9) 426.

66 Te Awa Tupua Act, s 12.

67 ibid.

68 See s 3; see also Tănăsescu, ‘Rights of Nature’ (n 19) 439–46.

69 See K Sanders, ‘“Beyond Human Ownership”? Property, Power and Legal Personality for Nature in Aotearoa New Zealand’ (2018) 30(2) JEL 207, 231–4; see also Macpherson and Ospina (n 19) 287; Tănăsescu, ‘Rights of Nature’ (n 19) 446.

70 See Kauffman and Martin, *Politics* (n 1) 218–19; see also ch 6.

71 The implementation strategy group, Te Kōpuka nā Te Awa Tupua, also includes representation for an energy-generating corporation and recreation interests, among others: see Te Awa Tupua Act, s 32(1).

72 In India and Bangladesh, the human right to a clean and healthy environment (derived from the right to life), see MS Islam and E O’Donnell, ‘Legal Rights for the Turag: Rivers as Living Entities in Bangladesh’ (2020) 23(2) APJEL 160, 166; in Colombia, biocultural rights recognising the rights of Indigenous communities on their traditional lands, which the Constitutional Court describes as ‘third generation human rights’, see Macpherson and Ospina (n 19) 291–2. Macpherson and Ospina are sceptical about the green normative potential of biocultural rights and suggest that they are broadly anthropocentric rather than ecocentric. Nevertheless, insofar as they recognise the integral connection between humans and nature, biocultural rights might be thought to support green norms and even offer an alternative to RoN. Evaluating this claim is beyond the scope of our analysis. However, as we go on to suggest in section 5, we do think that it would be worth examining whether the holistic understanding of values underpinning biocultural and other third-generation constitutional rights may be better captured by duty-based legal frameworks.

73 In India, the Uttarakhand State government challenged the decision, which has been indefinitely stayed by the Supreme Court: *State of Uttatrakhand & Ors. Versus Mohd. Salim & Others*, Special Leave to Appeal C No 16879/2017 (16 December 2019). In Bangladesh, the court of first instance sought to make the regulatory authority’s recommendations under existing legislation mandatory and judicially enforceable, but this was reversed on appeal: see Islam and O’Donnell (n 72) 174–6.

74 See Macpherson (n 40) 157–9; Macpherson and Ospina (n 19) 289–91.

75 See VAJ Kurki, *A Theory of Legal Personhood* (OUP 2019) ch 1; H Kelsen, *General Theory of Law and State* (Transaction 2006) 93.

76 See eg Kurki, *A Theory of Legal Personhood* (n 75) ch 3 (‘bundle theory’ of legal personhood).

77 See eg J Feinberg, ‘The Rights of Animals and Unborn Generations’ in WT Blackstone (ed), *Philosophy and Environmental Crisis* (University of Georgia Press 1974); T Regan, ‘Feinberg on What Sort of Beings Can Have Rights’ (1976) 14 Southern Journal of Philosophy 485; VAJ Kurki, ‘Can Nature Hold Rights? It’s Not as Easy as You Think’ (2022) 11 TEL 525; P Baard, ‘Are Rights of Nature Manifesto Rights (And Is That a Problem)?’ (2023) 29 Res Publica 425.

78 Kurki, ‘Can Nature Hold Rights?’ (n 77) 529; Baard (n 77).

79 See eg HLA Hart, ‘Bentham on Legal Rights’ in AWB Simpson (ed), *Oxford Essays in Jurisprudence* (OUP 1973).

80 See eg DN MacCormick, ‘Rights in Legislation’ in PMS Hacker and J Raz (eds), *Law, Morality, and Society: Essays in Honour of HLA Hart* (Clarendon Press 1977); M Kramer, ‘Rights Without Trimmings’ in H Steiner, M Kramer and NE Simmonds (eds), *A Debate Over Rights* (OUP 1998).

81 Although we do not deny the possibility of modifying the will theory to accommodate attributing rights to natural entities where control can be exercised on the entity’s behalf: cf Hart (n 79) 192, fn 86. See also n 87 below.

82 Stone, for example, writes that a natural entity that is afforded a right is recognised as ‘being a *beneficiary* in its own right’ (emphasis added): Stone (n 14) 480.

83 Feinberg (n 77).

84 Raz, *Morality of Freedom* (n 5) 177–8.

85 Kurki, ‘Can Nature Hold Rights?’ (n 77); Kramer, ‘Rights Without Trimmings’ (n 80). Kramer’s view is more nuanced than the preceding discussion might indicate. He acknowledges that non-sentient objects could in principle have rights under an interest theory but makes a separate moral argument against this expansion: M Kramer, ‘Getting Rights Right’ in M Kramer (ed), *Rights, Wrongs and Responsibilities* (Palgrave 2001).

86 Kurki, ‘Can Nature Hold Rights?’ (n 77) 550.

87 Although some recent work in RoN scholarship has highlighted the possibility, accommodated in certain Indigenous worldviews, that nature can (and does) express its needs and interests: see eg E Boulot and J Sterlin, ‘Steps Towards a Legal Ontological Turn: Proposals for Law’s Place Beyond the Human’ (2021) TEL 13, 28–31.

88 See eg Boyd, *Rights of Nature* (n 15) 103–4.

89 Stone (n 14) 464–5.

90 See eg R Routley, ‘Is There a Need for a New, an Environmental Ethic’ in *Proceedings of the XVth World Congress of Philosophy* (Sofia Press 1973) 205; Regan, ‘Feinberg’ (n 77); T Regan, ‘The Nature and Possibility of an Environmental Ethic’ (1981) 3 Environmental Ethics 19.

91 This is a traditional point of tension between animal liberationists and environmental ethicists: see eg JB Callicot, ‘Animal Liberation’ (1980) 2(4) Environmental Ethics 311.

92 cf Kurki, ‘Can Nature Hold Rights?’ (n 77) (the ‘anything goes’ approach).

93 ibid 551; Kurki, *Personhood* (n 75) 147.

94 Kurki, ‘Can Nature Hold Rights?’ (n 77); Kurki, *Personhood* (n 75) ch 4.

95 Kurki, ‘Can Nature Hold Rights?’ ibid 551.

96 ibid; Kurki, *Personhood* (n 75) 147.

97 cf Stone (n 14) 487.

98 On indirect models, see n 47 and accompanying text; cf Kurki,‘Can Nature Hold Rights?’ (n 77). In section 5, we argue that the governance paradigm offers a better understanding of these guardianship arrangements.

99 See Weis, ‘Environmental constitutionalism’ (n 13); see also L Bhullar, ‘Environmental Constitutionalism and Duties of Individuals in India’ (2022) 34(3) JEL 399; A Hellner and Y Epstein, ‘Allocation of Institutional Responsibility for Climate Change Mitigation’ (2023) 35(2) JEL 207; BJ Preston, ‘The Nature, Content and Realisation of the Right to a Clean, Healthy and Sustainable Environment’ (2024) 36(2) JEL 159.

100 Stone (n 14) 487.

101 See WN Hohfeld, *Fundamental Legal Conceptions as Applied in Judicial Reasoning and Other Essays* (W Cook ed, Yale UP 1919).

102 art 72.

103 Stone (n 14) 458.

104 See eg O’Donnell, ‘Rivers as Living Beings’ (n 40) (discussing the Birrarung/Yarra River and the Martuwarra/Fitzroy River in Australia, which are recognised as ‘living entities’ but not as ‘legal persons’). As we discuss further in section 5, the recognition of ecosystems as living entities in the two New Zealand Acts is far more important to how they advance green normativity than recognition of legal personality.

105 Stone (n 14) 488.

106 We borrow this language from VAJ Kurki, ‘Interest Theory’ in M Gilbert, G Sreenivasan and J Helmreich (eds), *Palgrave Handbook on Philosophy of Rights* (Palgrave, forthcoming) (manuscript also on file with the authors), who himself takes it from J Bowen, ‘The Interest Theory of Rights at the Margins: Posthumous Rights’ in M McBride and VAJ Kurki (eds), *Without Trimmings: The Legal, Moral, and Political Philosophy of Matthew Kramer* (OUP 2022).

107 According to non-justificatory accounts, an individual has a legal right just in case there is some legal duty that serves that individual’s interests in some way; see eg Bowen (n 106) 55.

108 Raz, *Morality of Freedom* (n 5) 166. For this reason, justificatory versions of the interest theory encounter difficulties explaining the existence of rights that seem primarily to be justified by their serving the interests of others (rather than the interests of the rights bearer): see eg J Raz, *Ethics in the Public Domain* (rev edn, OUP 2001) ch 3; R Cruft, ‘Against Individualistic Justifications of Property Rights’ (2006) 18 Utilitas 154; R Mullins, ‘Rights, Roles and Interests’ (2019) 16 Journal of Ethics and Social Philosophy 95; L Wenar, ‘The Nature of Claim Rights’ (2013) 123 Ethics 202.

109 Non-justificatory accounts are not necessarily incompatible with this ‘dynamic’ aspect of rights—a point stressed by Kramer in response to Raz: Kramer, ‘Rights Without Trimmings’ (n 80) 41–9. But justificatory accounts attribute this dynamic function to right as a matter of necessity.

110 As Raz observes, many legal rights have a ‘doubly harmonious function’, protecting individual interests only in virtue of the fact that doing so also serves the common good: Raz, *Ethics in the Public Domain* (n 108) 55.

111 See further Raz, *Morality of Freedom* (n 5) 198–203.

112 ibid 202.

113 See eg Birrell and Matthews (n 32) 282–4.

114 See n 19 and accompanying text; see also O’Donnell and others (n 9) 409–10. Although this ‘nature trumps’ view reflects a kind of philosophical confusion, we think that the confusion is understandable given the individualising tendencies of rights-based frameworks.

115 See eg A Arstein-Kerslake and others, ‘Relational Personhood: A Conception of Legal Personhood with Insights from Disability Rights and Environmental Law’ (2021) 30 GLR 530; Cano-Pecharroman and O’Donnell (n 40); Tănăsescu, *Understanding* (n 19) 57, 101, 132; Tănăsescu, ‘Rights of Nature’ (n 19) 448–53. cf J Nedelsky, *Law’s Relations* (OUP 2012).

116 Hohfeld (n 101).

117 Kurki, *Personhood* (n 75) 528–9 (emphasis added).

118 Constitution of Ecuador, arts 71, 74.

119 E Alston, ‘Ecuador’s 2008 Constitution: The Political Economy of Securing an Aspirational Social Contract’ (2018) 3 Constitutional Studies 69, 79.

120 See arts 26, 32, 34, 37–50.

121 Alston (n 119) 79.

122 Kauffman and Martin, *Politics* (n 1) 87; Kauffman and Martin, ‘Development’ (n 48) 134.

123 Kauffman and Martin, ‘Norms’ (n 48). This study only examines a selection of successful, ecocentric norm-driven civil society cases: 367.

124 Kauffman and Martin, *Politics* (n 1) 97–103; Kauffman and Martin, ‘Development’ (n 48) 136–8.

125 Kauffman and Martin, ‘Development’ (n 48) 133, 136–7, 140 fn 5.

126 ibid 134–6; Kauffman and Martin, ‘Norms’ (n 48) 379–93; Tănăsescu and others (n 48) 12. The first successful RoN litigation was brought by American landowners to protect interests that could be characterised as private property rights: see Kauffman and Martin, ‘Development’ (n 48) 136.

127 Kotze and Calzadilla (n 33) 427–9; see also citations in n 126.

128 See Kauffman and Martin, ‘Norms’ (n 48) 394; Kotze and Calzadilla (n 33) 425–27; Tănăsescu, ‘Rights of Nature’ (n 19) 435; Tănăsescu and others (n 48) 10.

129 Kauffman and Martin, ‘Norms’ (n 48) 369–70. The holistic implications may be overstated: this idea reflects the principle of harmonious construction, which requires courts to reconcile textual inconsistencies so far as possible.

130 Tănăsescu and others (n 48) 16.

131 We acknowledge that there are other, less common, adjudicative approaches that treat rights as ‘trumps’ rather than as protecting interests to be weighed or balanced, and that US constitutional rights jurisprudence is, at least on some views, a prominent example. However, a ‘rights as trumps’ approach fares worse from a green constitutionalist perspective, amplifying the general tendency of rights-based frameworks to characterise the interests of rights bearers as in competition with the public interest: see R West, ‘Rights, Harms, and Duties: A Response to *Justice for Hedgehogs*’ (2010) 90 BU LRev 819.

132 See R Dixon and LK Weis, ‘Constitutional Duties and the Judicial Role’ (2023) 2 PL 11; LK Weis ‘The Constitutional Office of the Legislature’ (2020) 70 UTLJ 214.

133 See Alston (n 119); see also Kauffman and Martin, *Politics* (n 1) 83; Kauffman and Martin, ‘Development’ (n 48) 132–3, 139.

134 See T Riofrancos, *Resource Radicals: From Petro-Nationalism to Post-Extractivism in Ecuador* (Duke UP 2020) ch 3. According to some scholars, the rights focus reflects the litigation-led priorities of US-based norm entrepreneurs: see eg Tănăsescu, ‘Rights of Nature’ (n 19) 436.

135 R Dixon and D Landau, *Abusive Constitutional Borrowing: Legal Globalization and the Subversion of Liberal Democracy* (OUP 2021) 76–7.

136 ibid 59, 74–80.

137 See also Tănăsescu, ‘Rights of Nature’ (n 19) 437–8; Tănăsescu and others (n 48) 12–13.

138 See discussion in section 3.

139 Raz, *Morality of Freedom* (n 5) 193–216.

140 Kauffman and Martin, *Politics* (n 1) 61–2.

141 See Cribb, Macpherson and Borchgrevink (n 40) 3; Kauffman and Martin, *Politics* (n 1) 72–3; Sanders (n 69) 213–21.

142 See Sanders (n 69) 222–30.

143 Cribb, Macpherson and Borchgrevink (n 40) 3; see also Kauffman and Martin, *Politics* (n 1) 75–6.

144 O’Donnell, ‘Living Beings’ (n 40) 650–1; O’Donnell and others (n 9) 421–5.

145 See Sanders (n 69) 211–13.

146 Kauffman and Martin, *Politics* (n 1) 158–9.

147 See O’Donnell, ‘Living Beings’ (n 40).

148 See Cano-Pecharroman and O’Donnell (n 40) 2. The same is true of US-based RoN ordinances: see Boyd *Rights of Nature* (n 15) 109–30; Kauffman and Martin, *Politics* (n 1) 63, 67, 68–70.

149 Tănăsescu and others (n 48) 14–15.

150 Riofrancos (n 134) ch 3.

151 See Kauffman and Martin, *Politics* (n 1) 219–22; Sanders (n 69) 213.

152 See Islam and O’Donnell (n 72) 162, 166; Kauffman and Martin, *Politics* (n 1) 203–6.

153 Kauffman and Martin, *Politics* (n 1) 203–4.

154 ibid 205.

155 O’Donnell and others (n 9) 421.

156 Sanders (n 69) 213; see also Tănăsescu, ‘Rights of Nature’ (n 19) 446–7.

157 Cano-Pecharroman and O’Donnell (n 40).

158 O’Donnell, ‘Living Beings’ (n 40) 659–61.

159 See Te Urewara Board, ‘Te Kawa O Te Urewere’ <www.ngaituhoe.iwi.nz/te-kawa-o-te-urewera> accessed 1 April 2025.

160 Cribb, Macpherson and Borchgrevink (n 40) 10, 11 (emphasis in original).

161 ibid 11.

162
*Centro de Estudios para la Justicia Social ‘Tierra Digna’ and Others v President of the Republic and Others* (*Atrato River case*) No T-622 of 2016, Corte Constitucional [Constitutional Court], Sala Sexta de Revision [Sixth Chamber] (Colombia) (10 November 2016) 137 (Tierra Digna).

163 See Macpherson (n 40) 157–9.

164 ibid 158–9, 159–60; see also Macpherson and Ospina (n 19) 290–1.

165 Macpherson (n 40) 153, 156.

166 See Kauffman and Martin, ‘Norms’ (n 48) 379–93.

167
*Municipality of Cotacachi v Ministry of Environment (Los Cedros case)* Judgment No 1149-19-JP/21, 10 November 2021, Constitutional Court of Ecuador, 13.

168 Kauffman and Martin, ‘Norms’ (n 48) 385; see also Tănăsescu and others (n 48) 8–9.

169 See JA McNeish and JM Socha, ‘Muddy Waters: On the Problematic Political Ecology of the Atrato Ruling, Colombia’ IJHR (publ online 6 March 2024) <https://doi.org/10.1080/13642987.2024.2324121>.

170 Entrenchment could be legal or political; we do not take a general position on which form is appropriate, as this issue is context dependent.

171 Although Ireland’s Constitution was the first to include directive principles, their adoption by India has been most influential in their adoption throughout the Global South: see LK Weis, ‘Constitutional Directive Principles’ (2017) 37(4) OJLS 916, 923–4.

172 ibid 919–23.

173 T Khaitan, ‘Constitutional Directives: Morally Committed Political Constitutionalism’ (2019) 82 MLR 603.

174 T Khaitan, ‘Guarantor Institutions’ (2021) 16(1) ASJCL S40, S53.

175 ibid S50–S51.

176 ibid S52.

177 ibid S53–S56.

178 ibid S47.

179 D Samararatne, ‘Sri Lanka’s Guarantor Branch: Constitutional Resilience by Stealth?’ in S Jhaveri, T Khaitan and Dinesha (eds), *Constitutional Resilience in South Asia* (Hart Publishing 2023) 257, 274–8.

180 Khaitan, ‘Guarantor Institutions’ (n 173) S58.

181 Kauffman and Martin, *Politics* (n 1) 65–6.

182 Raz, *Morality of Freedom* (n 5) 210. For a different account of the primacy of obligations in the ecological context, which is based on facts about holistic social ontology, see Birrell and Matthews (n 32) 285 (arguing that obligations are derived from facts about interdependency).

183 ibid. ‘Intrinsic’ here means having non-instrumental value.

184 ibid.

185 ibid 211–13.

186 ibid 40–2, 212.

187 See Routley (n 90) 207.

188 ibid 208.

189 ibid.

190 Cribb, Macpherson and Borchgrevink (n 40) 10, 11.

191 Constitution of Ecuador, ss 71, 72, 73.

192 As observed above (n 72), we think that biocultural rights are one example. Others include protection for cultural heritage, Indigenous peoples and religion.

193 Kramer, for example, argues that all duties correlate with at least one right, and proposes that this thesis resists empirical refutation: Kramer, ‘Rights Without Trimmings’ (n 80) 24; see also K Barker, ‘Private Law, Analytical Philosophy and the Modern Value of Wesley Newcomb Hohfeld: A Centennial Appraisal’ (2018) 38 OJLS 585, 599–600.

194 See eg Kramer, ‘Rights Without Trimmings’ (n 80) 59 (suggesting that all public duties not owed to individuals are owed to the state).

195 cf Tonja Jacobi, ‘The role of theory in empirical legal studies’ (2023) 1(2) Comparative Constitutional Studies 222.

196 See Eco Jurisprudence Monitor (n 39).

